# Curvelet-enhanced transformer architecture for blurred action fine-grained detection

**DOI:** 10.1038/s41598-025-33985-6

**Published:** 2025-12-31

**Authors:** Yuxiang Ren, Zhetao Guo, Wei Zhang, Yushi Shen, Ying Xing

**Affiliations:** 1 Beijing Dianjing Ciyuan Network Technology Co.,Ltd, Beijing, 100124 China; 2 Cloudspace Technology Co., Ltd, Beijing, 100176 China; 3 The Third Affiliated Hospital, Beijing University of Chinese Medicine, Beijing, 100029 China; 4 NovNet Computing System Tech Company, Beijing, 100081 China; 5 School of Intelligent Engineering and Automation, Beijing University of Posts and Telecommunications, Beijing, 100876 China

**Keywords:** Motion blur restoration, Multi curvelet, Transformer, Human behavior recognition, Engineering, Applied mathematics, Computer science

## Abstract

This study proposes a novel Multi Curvelet Transformer Network (MCTN) for fine-grained human behavior recognition in dynamic video scenarios. A key challenge in this field lies in accurately identifying human actions under adverse conditions such as motion blur, occlusion, and varying illumination. To address this, we introduce a motion blur restoration module leveraging the curvelet transform to enhance motion image clarity, thereby improving downstream behavior detection. Furthermore, we enhance the Transformer architecture by embedding curvelet-based multi-scale attention mechanisms, which significantly improve the model’s ability to extract spatial-temporal features at different resolutions. The proposed network also adopts a multi-curvelet transform structure to deepen semantic representation. Experimental results on benchmark datasets, including an action recognition dataset and the MSCOCO dataset, demonstrate that MCTN achieves superior performance, reaching a mean average precision (mAP) of 0.822. These results underscore the potential of MCTN in real-time intelligent video analysis and human-computer interaction applications.

**Supplementary Information:**

The online version contains supplementary material available at 10.1038/s41598-025-33985-6.

## Introduction

Accurate recognition of human behavior states in dynamic environments is essential for a wide range of intelligent video applications, including sports performance analysis, human–computer interaction, and surveillance. With the rapid development of image processing and computer vision technologies, it has become feasible to analyze and interpret human postures and actions from video data with increasing precision^1–5^. Such capabilities not only facilitate performance monitoring but also enable targeted guidance in both professional training and automated systems.

However, human behavior recognition in high-speed and complex environments remains a significant challenge^6–8^. Fast-paced actions such as rapid gestures, sudden turns, and subtle limb movements often occur within fractions of a second, making them difficult to capture and classify accurately. Moreover, the variability in individual motion patterns, including unconventional postures and spontaneous reactions, cannot be effectively handled by rigid rule-based methods. External environmental factors—such as dynamic lighting conditions, motion blur, occlusions, and camera noise—further degrade the reliability of visual features^9–11^. Additionally, in scenarios where multiple subjects or objects (e.g., players and sports equipment) interact simultaneously, real-time tracking and behavior recognition become even more complicated due to frequent occlusion and spatial-temporal interference.

Another critical limitation lies in the availability and quality of annotated datasets. For niche activities or fine-grained motion analysis, labeled data often require expert involvement, leading to high annotation costs and limited sample sizes^12,13^. These constraints highlight the urgent need for robust models capable of handling complex visual scenes with limited supervision, adaptable to various dynamic human activities.

Around these difficulties, player behavior detection^14–18^ has been proposed one after another. Nicolai et al.^19^ proposed the DeepSORT algorithm, which is a strategy for object tracking by the object detection. It uses the Hungarian algorithm to associate the tracking box and the detection box, fuses the appearance information and the Mahalanobis distance to obtain the best matching value, and then predicts the position of the tracking box at the next moment according to the Kalman filter. Finally, it decides whether to update the detection box according to the result and the strategy. Wu et al.^20^ used a residual convolutional neural network to estimate the continuous 2D upper body pose of a table tennis player and then used a recurrent long short-term memory network to learn the serving motion of the player and predict the landing point of the table tennis player. Huang et al.^21^ used OpenPose as a mankindkey point detection to recognize posture, and corrected the exercise training elements through the index scores to reduce the sports injuries of athletes. Aiming at the blurring phenomenon of acquired images caused by high-speed motion, the MixSort tracker proposed by Cui et al.^22–24^ is based on ByteTrack, OC-SORT, and MixFormer for end-to-end connection. However, despite the performance improvement, the algorithm still faces the challenges of real-time and computational resource consumption, especially when dealing with complex scenes and a large number of targets. Han et al.^25^ suggested a combined asymmetric net and triple loss function of the tracking, which can prove the effectiveness of the complex moving object in the guarantee and the number of cases.

These methods have achieved certain results in the field of athlete behavior state detection research, and they have high accuracy for the state recognition of a single frame. However, complex environments and large motion actions need to solve image problems such as motion blur. To solve these problems, we propose a Multi Curvelet Transformer Network for Athlete Behavior Detection. The main contributions of this paper are as follows:

1. We propose a curvelet transformer-based motion blur restoration method, which exploits the relation between consecutive video frames to compensate for the information lost due to motion blur.
2. By incorporating curvelet transform into the self-attention mechanism, we deepen the understanding of the relationship between video content and improve the accuracy of action detection. At the same time, we also innovatively design a multi-curvelet transform structure, which can capture image information at different scales and deeply mine the deep semantic features of images.

## Related works

Recent advancements in athlete behavior state detection have seen significant contributions, especially in the field of human pose detection. In the domain of 2D state detection, Wang et al.^26^ proposed an hourglass structure that adaptively extracts features at multiple scales to better accommodate diverse human poses. While this approach is promising, it struggles with high computational complexity during the convolution process, despite efforts to mitigate this through downsampling and upsampling operations. Chen et al.^27^ utilized a multi-scale pyramid method, dividing the image into various scales and processing them separately. Although this method improves accuracy by handling different scales individually, it suffers from the limitations of static feature extraction and does not account for dynamic changes in the athlete’s movement. Sun et al.^28^ introduced HRNet, a model that retains high-resolution feature information across multiple resolutions to improve the accuracy and speed of attitude detection. However, while HRNet excels at capturing detailed feature maps, it may face challenges in real-time processing, especially in highly dynamic environments such as sports. Xu et al.^29^ proposed a multi-person pose detection system that first detects the object in the input image, isolates the human body, and then applies a separate network for keypoint detection. While this method is effective in handling multiple subjects, it requires robust handling of occlusion and interaction between players, which may not be adequately addressed in some cases.

In the field of 3D state detection, Ji et al.^30^ demonstrated the feasibility of using a deep neural network (CNN) to directly predict 3D human pose with acceptable accuracy. However, despite the network’s ability to predict 3D coordinates, the method struggles to generalize across complex and varying postures, particularly in the context of fast-paced athletic movements. Heravi et al.^31^ employed a combined model of CNNs and RNNs to learn both the spatial structure of human poses and the relative positions of joints. While these hybrid methods offer an improvement in capturing temporal information^32–34^, it can still be computationally intensive, making it less suitable for real-time applications in sports. Jiang et al.^35^ introduced a domain-based 3D human pose distribution model that predicts more diverse human poses with greater complexity. However, despite its capacity to predict a variety of poses, this method can suffer from inaccuracies in predicting poses with extreme angles or fast movements, which are typical in sports environments.

While recent techniques have achieved notable success in athlete behavior detection^36,37^, they still exhibit critical limitations when deployed in fast-paced scenarios such as niche sports. In particular, applications to sports like pickleball are challenged by frequent motion blur, rapid temporal transitions, and multi-object occlusion, which significantly degrade model performance. Moreover, the scarcity of labeled training data in such domains—where annotations often rely on domain experts—further restricts the effectiveness of data-driven approaches and hinders generalization.

## Methods

**Fig. 1:**
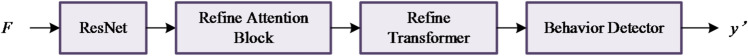
The framework of MCTN for athlete behavior detection.

Aiming at the serious motion blur problem in motion images, we propose a multi curvelet Transformer network for athlete behavior detection method. To deal with the challenge of image blur during sports, we specially design the motion blur recovery (MBR) module, and refine the internal structure of the Transformer. This innovative design enables our model to effectively detect the behavior of blurred samples. An overview of the entire network is present in Fig. 1.

### Motion blur restoration module based on curvelet transform

For any image *F* of a player, we first decompose it into a series of video frames, and represent these video frames by {*f*_*i*_} to form a video sequence. For the blurred frames, a homography model based on warp bundling is used to register multiple adjacent video frames to the blurred frame. To improve the registration, we employ the block method in the process of calculating the registration images of blurred video frames and their adjacent video frames. Specifically, we divide each video frame into several uniform blocks, and let the number of blocks in each video frame be *Q*, *I*_*i*,*q*_ is a block in the video frame *f*_*i*_, where *q* ranges from [1, *Q*]. To obtain the homography *G*_*i, n,q*_, we use a warping-based motion model, which is described as Eq. (1):

1 $$\:{\widehat{C}}_{i,n,q}={G}_{i,n,q}\bullet\:{C}_{i,n,q}$$

where $\:{\widehat{C}}_{i,n,q}$ and *C*_*i, n,q*_ represent the position coordinates from image block *I*_*i, n,q*_ to block *I*_*i*,*q*_ before and after warping respectively, and the value range of *n* is [0, *N*], where *N* denotes the account of adjacent video frames waiting for registration. The formula for calculating the registered image is Eq. (2):

2 $$\:{I}_{i,n,q\to\:i,q}={G}_{i,n,q}\bullet\:{I}_{i,q}$$

where *G*_*i, n,q*_ is further defined as the trainable homography, which represents the registration process from image block *I*_*i, n,q*_ to block *I*_*i*,*q*_, denoted as *I*_*i, n,q→i, q*_. In particu*lar*, when *n* = 0,* I*_*i, n,q→i, q*_​ represents the image block *I* itself. The adjacent video frame *f*_*i, n,q→i, q*_ after registration is composed of all the registered image blocks *I*_*i, n,q→i, q*_, where the value of *q* is in the range [1, *Q*].

Equations (1) and (2) describe how each block of an adjacent video frame is geometrically warped to align with the corresponding block in the blurred reference frame. Conceptually, this can be understood as “shifting and stretching” small image patches so that overlapping structures (e.g., edges of limbs or equipment) match across frames. This alignment reduces inconsistencies caused by rapid motion. Once registered, the frames are processed in the frequency domain using the curvelet transform. Unlike the Fourier transform, which decomposes signals into sinusoidal waves, the curvelet transform provides multi-scale, multi-orientation representations, making it particularly adept at capturing directional features such as edges and contours.

Then, we process the registered video frames in the frequency domain. Given the continuous and large spatial span characteristics of motion actions, we introduce curvelet transform into MBR. The curvelet transform shows superior performance over the traditional transform in capturing edges and other exotic features. The curvelet transform contains three key parameters: scale (*s*), orientation (*o*) and position (*p*), which are used to accurately describe the characteristics of the transform. The basis function of the curvelet transform can be expressed as *δ*_*s*,*o*,*p*_. The construction of the curvelet transform is based on the radial window function and angle window function. The radial window function, designated as µ(w), is defined over the domain *w* ∈ [1/2, 1], while the angular window function, denoted as ν(*x*), operates within the domain *x* ∈ [− 1, 1]. Both functions must adhere to specific mathematical constraints, as outlined in Eqs. (3–4).However, since the frames of an image are discontinuous in both time series and pixel space, it is necessary to further convert them into discrete functional forms. Then, the curvelet transform is performed on the registered video frames to obtain the discrete curvelet coefficients $\:\partial\:{\prime\:}\left(s,o,p\right)$. The method of updating these coefficients is described by Eq. (9).

3 $$\:{\partial\:}_{{f}_{i}}^{{\prime\:}}\left(s,o,p\right)=\sum\:_{n=0}^{N}{W}_{{f}_{i}}\left(s,o,p\right){\delta\:}_{s,o,p}\left({y}_{{f}_{i}}\right)$$

where$\:{\partial\:}_{{f}_{i}}^{{\prime\:}}\left(s,o,p\right)$ denotes the discrete curvelet coefficient of video frame *f*_*i*_. $\:{\delta\:}_{s,o,p}\left({y}_{{f}_{i}}\right)$ denotes the continuous curvelet coefficient of video frame *f*_*i*_ at position *y*, and $\:{W}_{{f}_{i}}\left(s,o,p\right)$ is the corresponding weight of the coefficient. The specific way to calculate the weight is given by Eq. (10).

4 $$\:{W}_{{f}_{i}}\left(s,o,p\right)=\frac{{e}^{{\delta\:}_{s,o,p}\left({y}_{{f}_{i}}\right)}}{\sum\:_{n=0}^{N}{e}^{{\delta\:}_{s,o,p}\left({y}_{{f}_{i}}\right)}}$$

where *i* in *f*_*i*_ is the set of low, middle and high frequency information of the image.

To facilitate understanding of the motion blur restoration pipeline, we provide a pseudo-code.

**Figure:**
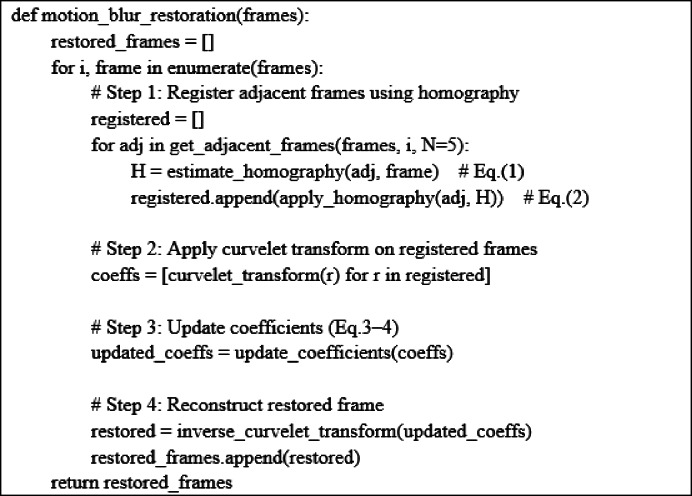
Algorithm 1: Pseudo-code for motion blur restoration using curvelet transform.

### Refining transformer with MBR

Using the MBR module, we optimize and upgrade the Transformer to effectively deal with motion blur in images.

**Fig. 2:**
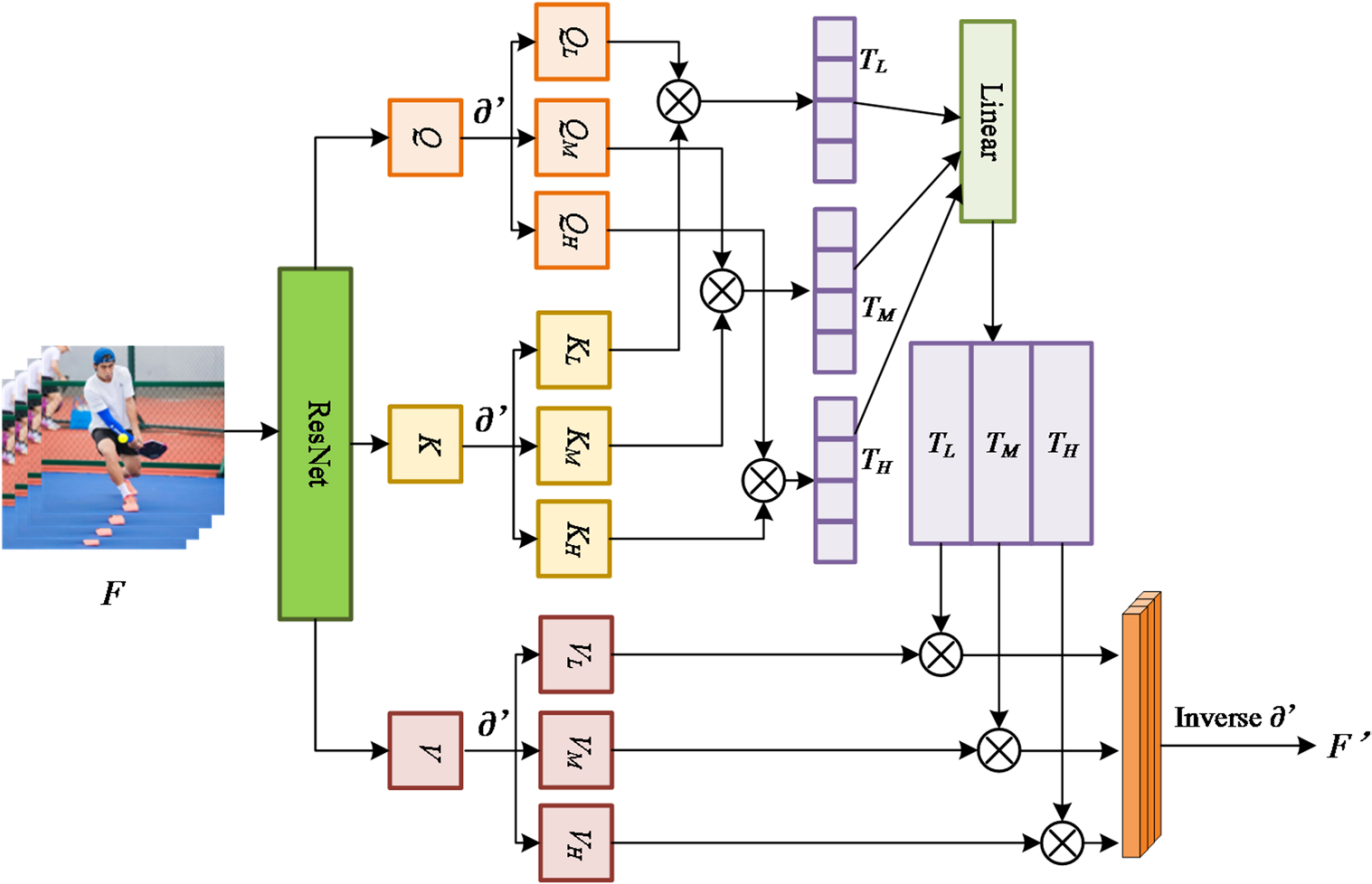
The framework of refined attention block with MBR (∂′).

We make a structural improvement to the self-attention mechanism by introducing MBR, as shown in Fig. 2. In this improved structure, we input the video frame F into the ResNet to obtain the corresponding features. Subsequently, these features are used as query (Q), key (K), and value (V), which are fed into the attention mechanism. The formula for the original self-attention mechanism (SA) is shown in Eq. (5) :

5 $$\:SA\left(F\right)=\frac{{e}^{\frac{\left({f}_{n}{W}^{Q}\right){\left({f}_{m}{W}^{K}\right)}^{T}}{\sqrt{d}}}}{{\sum\:}_{k=1}^{n}{e}^{\frac{\left({f}_{n}{W}^{Q}\right){\left({f}_{p}{W}^{K}\right)}^{T}}{\sqrt{d}}}}{f}_{j}{W}^{V}$$

**Fig. 3:**
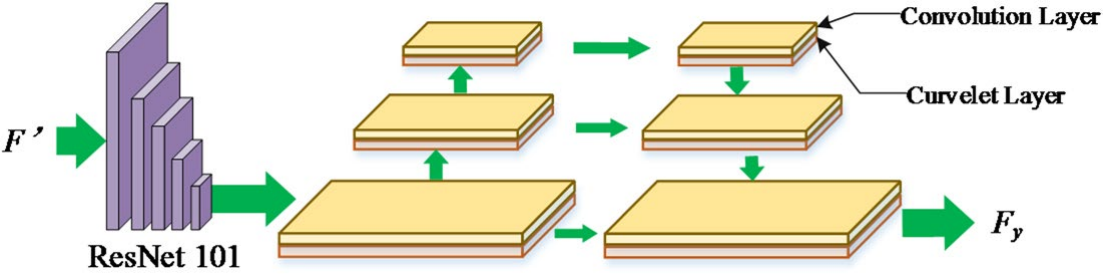
The framework of multi curvelet transformation.

Our process of using MBR to improve self-attention is shown in Eqs. (6–8):

6 $$\:{f}_{i}{\prime\:}=\partial\:{\prime\:}\left({f}_{i}\right)$$

7 $$\:{\sigma\:}_{i,j}=\frac{\left({f}_{i}{\prime\:}{W}^{Q}\right){\left({f}_{i}{\prime\:}{W}^{K}\right)}^{T}}{\sqrt{d}}$$

8 $$\:{T}_{M}={e}^{{\sigma\:}_{i,j}}/{\sum\:}_{k=1}^{n}{e}^{{\sigma\:}_{i,j}}\bullet\:{f}_{i}{\prime\:}{W}^{V}$$

where M refers to a set that contains information in low, middle and high frequency. To integrate and utilize these features from different frequency bands, we adopt the strategy of concatenation and apply the inverse curvelet transform to the concatenation results, as shown in Eqs. (9–10) :

9 $$\:T=W\left(\left[{T}_{L},{T}_{M},{T}_{H}\right]\right)$$

10 $$\:{F}^{{\prime\:}}=Inverse\left(T\right)$$

where *W* is a trainable matrix, [,] denotes the concatenation of features, and *Inverse*() is the inverse curvelet transform calculation. We fully optimize the Transformer architecture by using MBR-improved self-attention (MSA). In addition, in the network architecture, we construct a Polycurvelet Transform (PCT) structure as shown in Fig. 3, which can deeply mine the content features in F_y_.

We apply MCTN to the motion image processing of players. The proposed MCTN can not only effectively remove image noise, but also solve the problem of motion blur. Finally, we embed MCTN into various transformer-based behavior detection models to ensure accurate and efficient evaluation of the actions of players.

Equations (13)–(15) describe the integration of frequency-domain information into the self-attention mechanism. The intuition is that standard attention treats all pixel-level features uniformly, whereas the proposed MBR-enhanced attention assigns different importance to low-, mid-, and high-frequency components. Low-frequency signals capture global shape, mid-frequency signals capture texture and contour information, and high-frequency signals capture fine edges. By concatenating these multi-frequency features (Eqs. 16, 17) and performing an inverse curvelet transform, the model reconstructs feature maps that retain both global consistency and fine structural details. This enhancement enables the Transformer to more robustly attend to motion-relevant regions, even under severe blur or occlusion.

**Figure:**
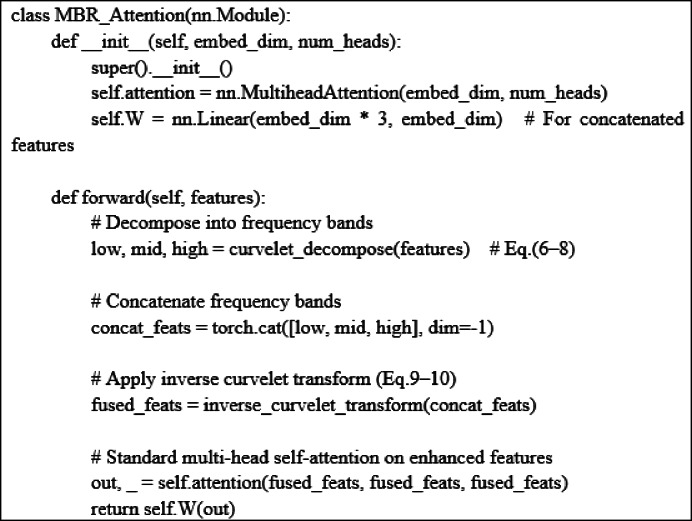
Algorithm 2: MBR-enhanced self-attention.

## Experiment and analysis

### Dataset and details

To evaluate the effectiveness of the proposed behavior state analysis framework, we conducted experiments on both domain-specific and general-purpose datasets. Specifically, we utilized the Pickleball Dataset (source) to assess the model’s performance in a specialized sports context characterized by rapid and irregular movements. This dataset serves as a representative case for challenging real-world scenarios involving motion blur and fine-grained athlete behavior recognition. In parallel, we employed the widely-used MS COCO dataset^38^ to validate the robustness and generalization capability of the model under diverse conditions. The COCO dataset includes annotations for 17 body keypoints per person, with over 1.5 million object instances spanning 80 object categories. It contains data from approximately 250,000 individuals, averaging 2 annotated persons per image, with some images featuring up to 13 individuals.

In the training pipeline, the model configuration parameters are shown in Table 1, covering the key elements of learning rate, training rounds, batch size, decay, and gradient descent. In the early stage of training, the model first uses the cross-entropy loss function for 20 rounds of basic training, aiming to obtain a relatively stable preliminary model. Subsequently, based on this, we further implemented 10 rounds of reinforcement learning training, aiming to deeply optimize the model through specific evaluation indicators. It is worth noting that the learning rate adopts a decreasing strategy during the training process. After every two rounds of training, the learning rate is reduced by 20% until the whole training process is completed. We adopt DETR^39^ as the baseline model for our method.

**Table 1:** Model parameter settings during training.

Parameters	Value
Initial learning rate	$\:2\times\:{10}^{-4}$
Training rounds	40
Runtime per epoch	20.2 min
Batch-size	20
Decay	0.85
Optimizer	SGD
Loss functions	Cross entropy
CPU	Ryzen 7 9700X
GPU	RTX 4090
Image input size	512 × 512
Image feature dimension	1024

In addition, to accurately evaluate the performance of MCTN, we use mAP (mean Average Precision) and OKS (Object Keypoint Similarity) as evaluation criteria, whose formulas are shown in Eqs. (11–13):

11 $$\:AP=P\times\:\sum\:_{n}\left({R}_{n}-{R}_{n-1}\right)$$

12 $$\:mAP=\sum\:_{i=1}^{N}A{P}_{i}$$

13 $$\:OKS=\frac{1}{N}\sum\:_{i=1}^{N}{v}_{i}{e}^{(-\frac{{d}_{i}^{2}}{2{\sigma\:}^{2}})}$$

where N refers to the total number of keypoints, *d* and *v* represent the Euclidean distance and visibility label of keypoints, respectively, and *σ* is a scale parameter.

### Parameter experiments

Before conducting ablation experiments and comparison experiments, we need to determine some parameters of the model. Our parameter experiments, including the number of adjacent video frames N, the number of encod-decoder layers M of Transformer, and the number of CNN + Curvelet block layers L of PCT, are all performed in the complete MCTN.

**Fig. 4:**
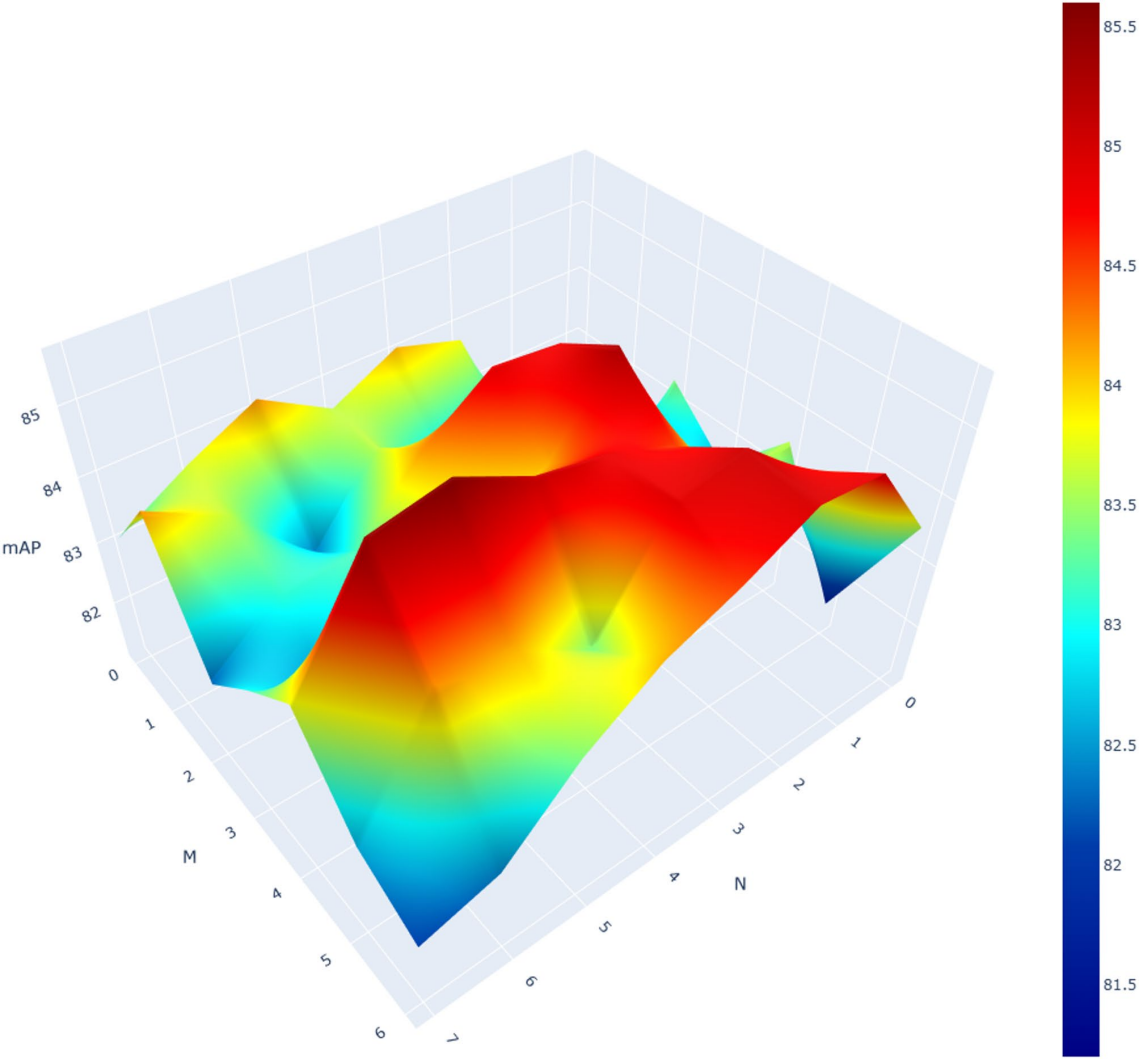
The measurement of parameters M and N.

Firstly, given that there is an interactive relationship between N and M, we decide to experiment on these two parameters simultaneously. The N parameter directly determines the number of relevant frames to be sampled, which has the influence on the curvelet transform. The M parameter is directly related to the output of MCTN. To ensure the accuracy of the parameter determination, we removed the PCT structure from the model during this experiment. The experimental results are reported in Fig. 4. After careful analysis, we find that when M is set to 6 and N is set to 5, the model can achieve the highest mAP of 0.856. This finding provides an important reference for us to optimize the model performance in the future.

**Fig. 5:**
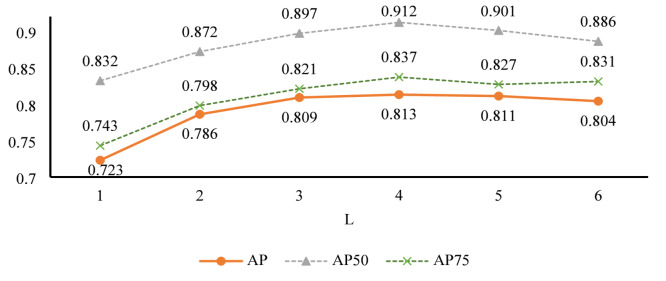
The choice of parameter L.

Subsequently, we determined the parameter L. In the experiment, we adopted the optimal configuration determined earlier, that is, M is set to 6 and N is set to 5. The results are presented in Fig. 5. Through observation, we find that when the value of L is 4, the performance reaches the best, and the specific performance is AP^50^, AP^75^ and AP achieve 0.813, 0.912 and 0.837, respectively. We repeated the experiment several times and made sure that the conditions of each experiment were as consistent as possible. The results demonstrate that the model is stable and excellent under the condition of L = 4, which proves the effectiveness of the parameter configuration.

### Ablative studies

To evaluate the effect of the three modules MBR, MSA, and PCT, ablative studies will be performed. Given that MBR is the basis of MSA and PCT, the specific setup of the experiment is shown in Table 2. In this table, we analyze the role of MBR in MSA and PCT in detail, and the results show that MBR improves the performance of MSA and PCT by 2.5% and 1.2% of AP scores, respectively. Further observed, MSA increased by 1.3% compared with the baseline model of AP scores, while PCT based on the MSA (w/MBR) of 1.2% of AP score again. Finally, when the three modules of MBR, MSA, and PCT are combined, the overall performance reaches the best, with the AP score of 0.813, AP^50^ score of 0.912, AP^75^ score of 0.837, AP^M^ score of 0.775 and AP^L^ score of 0.822.

**Table 2:** Ablation experiments on pickleball dataset.

Methods	AP	AP^50^	AP^75^	AP^M^	AP^L^
Baseline	0.751	0.846	0.795	0.741	0.773
+MSA (w/o MBR)	0.764	0.864	0.799	0.742	0.786
+MSA (w/MBR)	0.789	0.869	0.812	0.756	0.798
++PCT (w/o MBR)	0.801	0.885	0.821	0.771	0.815
++PCT (w/MBR)	0.813	0.912	0.837	0.775	0.822

As a base module, MBR is effective in the performance enhancement of subsequent modules. By introducing multi-scale and multi-direction curvelet transform, MBR enhances the model’s ability to capture image features, and provides a richer information basis for the subsequent self-attention mechanism and Transformer. By improving the self-attention mechanism, MSA effectively uses the features provided by MBR, thereby improving the AP score, indicating that MSA can more accurately capture the key information and increase the accuracy of behavior detection. PCT further improves the AP score by 1.2%. This is due to the polycurvelet transform structure, which can deeply mine the deep semantic features of images at different scales. When the three modules of MBR, MSA and PCT are used together, the performance reaches the best, indicating that these three modules complement each other in function and jointly optimize the behavior detection. MBR provides a wealth of multi-scale and multi-direction features. MSA effectively utilizes these features by improving the self-attention mechanism. PCT further mines the deep semantic information of images. The collaborative work of these three modules enables the model to achieve significant improvement in multiple evaluation indicators.

### Comparisons with state-of-the-art models

We compared the performance of our method with the SOTA (State-of-the-Art) methods, including SwinT^40^, SimpleBaseline^41^, DERK^42^, HigherHRNet + SWAHR^43^, AECA^44^, EBA^45^, TokenPose^46^, RIFormer^47^, TransPose^48^, HRNet^28^, PRTR^49^, BCIR^50^, and SimCC^51^. To verify the broad applicability and extensibility of our method, experiments are implemented on the Pickleball Dataset and MSCOCO dataset respectively.

#### Comparisons on the pickleball dataset

First, we evaluate the performance of MCTN on the Pickleball Dataset. According to the data in Table 3, MCTN shows excellent performance, with an AP score of 0.813, an AP^75^ score of 0.837, an AP^M^ score of 0.775, and an AP^L^ score of 0.822, which surpasses all the methods participating in the comparison. Furthermore, we optimized and upgraded the backbone of MCTN by replacing the original DERT with the more advanced RT-DETRv3^52^. This change resulted in significant performance improvements, with the AP score jumping to 0.822, AP^75^ to 0.846, AP^M^ to 0.780, and AP^L^ to 0.844. This result strongly proves that MBR, MSA and PCT modules have excellent plug-and-play characteristics, which can be easily integrated into different backbones to improve performance. In MCTN, MBR enhances video frame clarity through deblurring algorithms, providing high-quality input for subsequent analysis. MSA strengthens the correlation of spatiotemporal features via multi-head parallel computation, enabling precise capture of athletes’ dynamic motion patterns. PCT, through multi-scale and multi-directional geometric decomposition, breaks down complex motions into directionally sensitive feature sub-bands, enhancing the representation of details such as limb rotations and rapid movements. Together, these three components achieve full-pipeline optimization, spanning blur restoration, feature refinement, and high-dimensional semantic extraction. In addition, we also deeply study the influence of frame input size on model performance. By comparing the performance results of models with different frame sizes, we find that appropriately increasing the frame size can further improve the model to a certain extent.

**Table 3:** Compare with methods on the pickleball dataset. In this experiment, we re-implement the SOTA methods on this dataset.

Methods	Backbone	Frame size	AP	AP^75^	AP^M^	AP^L^
SwinT	SwinT	256 × 192	0.755	0.795	0.723	0.801
SimpleBaseline	ResNet-50	256 × 192	0.748	0.768	0.704	0.781
DERK	HRNet-W32	512 × 512	0.751	0.776	0.743	0.802
HigherHRNet + SWAHR	HRNet-W32	512 × 512	0.786	0.799	0.771	0.793
AECA	ResNet-18	384 × 288	0.769	0.787	0.762	0.810
EBA	ResNet-18	256 × 255	0.799	0.821	0.756	0.809
TokenPose	TokenPose-L/D24	256 × 192	0.796	0.819	0.751	0.810
RIFormer	HRFormer-B	256 × 192	0.801	0.818	0.764	0.813
MCTN	DETR	256 × 192	0.813	0.837	0.775	0.822
MCTN	RT-DETRv3	256 × 192	0.822	0.841	0.778	0.846
MCTN	DETR	384 × 288	0.816	0.837	0.777	0.827
MCTN	RT-DETRv3	384 × 288	0.822	0.846	0.780	0.844

**Fig. 6:**
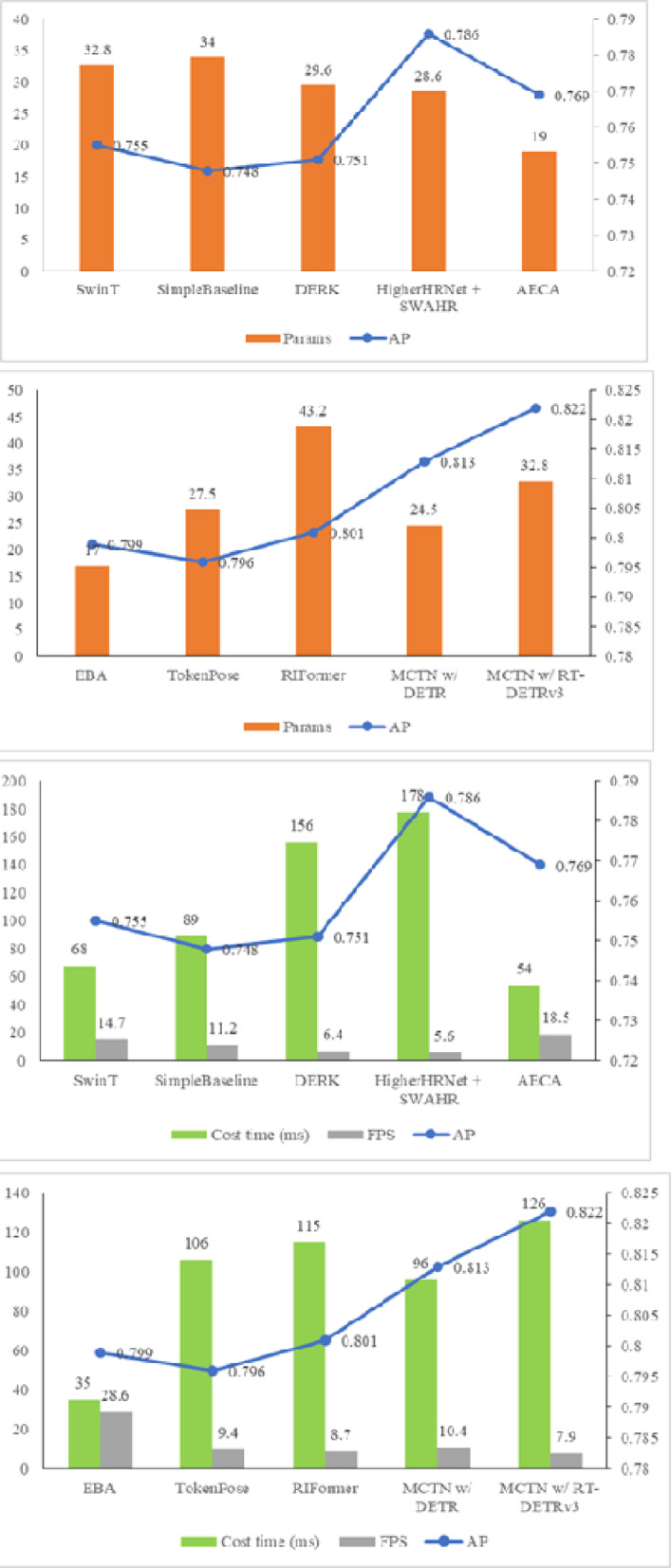
Comparison with other methods on the Pickleball dataset in terms of cost time FPS and parameters.

Secondly, we conduct a comparative analysis of the running time, frame per second (FPS) and the parameters of the MCTN model, and the results are shown in Fig. 6. At the same time, Fig. 6 also visually shows the performance of each model in the form of a line chart. It can conclude that the model of MCTN(w/ DETR) has 24.5 M parameters and the execution time is 96ms (10.4 FPS). The MCTN(w/ RT-DETRv3) model has more parameters, reaching 32.8 M, and its running time is a little longer, 126ms (7.9 FPS). Compared with other methods, it can be concluded that the improvement of the performance of MCTN depends not only on the increase of the parameters and running time, but also on the rationality of the model structure design and the effectiveness of the algorithm optimization. By introducing innovative modules such as MBR, MSA and PCT, MCTN realizes the efficient capture and accurate analysis of the key information of the image, to achieve performance improvement while maintaining low computational complexity.

**Fig. 7:**
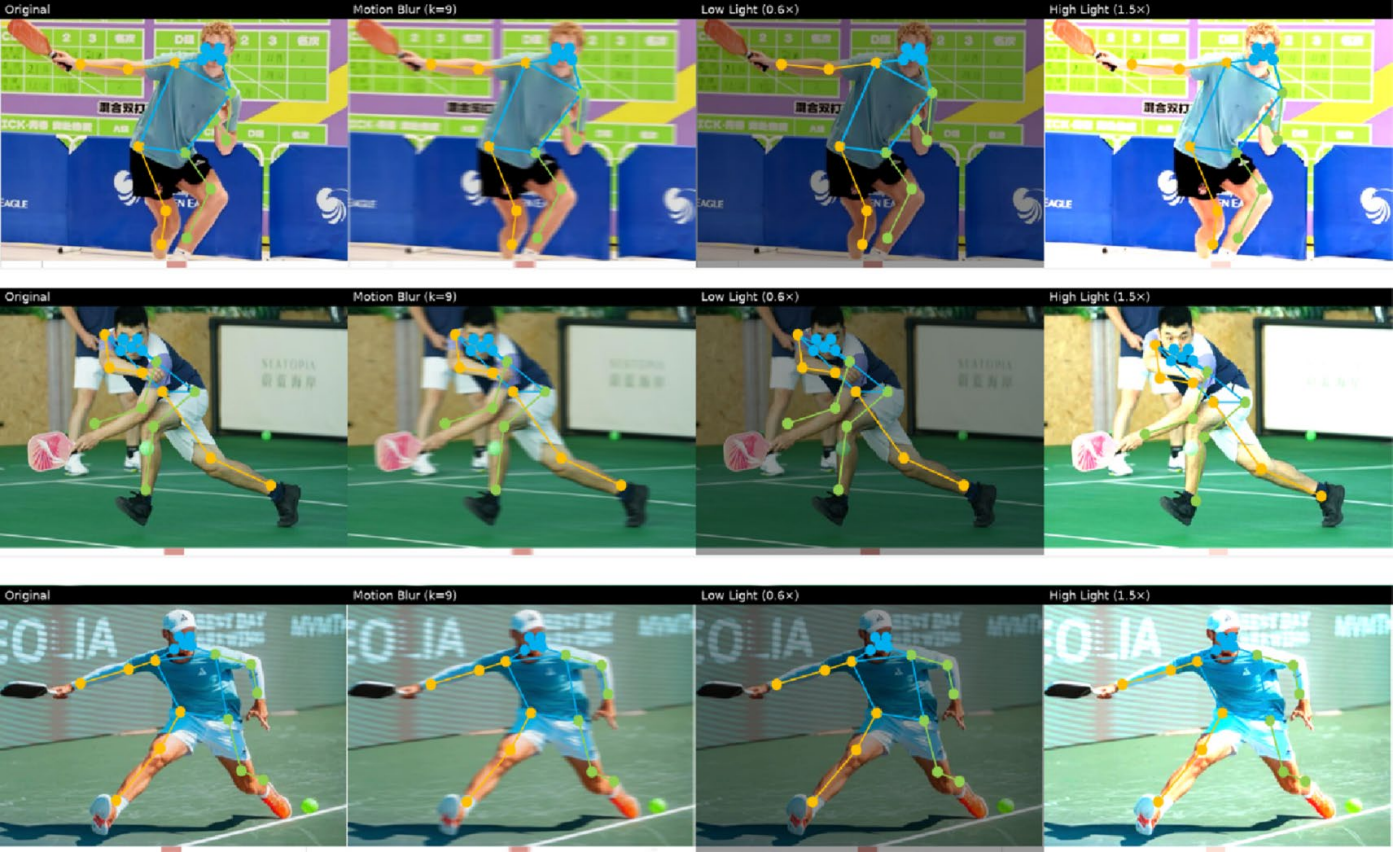
Visualization of MCTN performance under different input conditions.

Finally, we present the behavior analysis results of player images in the form of visualization, as shown in Fig. 7. For each athlete, the top row shows the input frames under four conditions—Original, Motion Blur, Low Light, and High Light—while the bottom row presents the corresponding pose estimation outputs generated by the proposed model.

It can be observed from the figure that MCTN can still stably and accurately capture the keypoints of the human body, such as shoulder, elbow, knee, etc., even when the athlete’s movement changes rapidly and the image appears to be blurred to a certain extent. This is crucial for subsequent applications such as behavior recognition, action analysis, and athlete training feedback. In the original and moderately degraded conditions, the model consistently captures the global body structure and accurately localizes major joints, ensuring reliable pose estimation. Under motion blur, the curvelet-based motion restoration and frequency-aware attention enable the network to preserve critical edge details, which mitigates the loss of high-frequency information and supports stable detection of limb orientations. Similarly, in both low-light and high-light environments, the multi-scale curvelet representation provides enhanced adaptability by balancing global shape cues and local textures, thereby reducing the negative impact of illumination imbalance. Thanks to its internal MSA and PCT, MCTN can capture subtle changes in human posture at different scales, and uses time series information to enhance the understanding of motion patterns, to effectively deal with the challenge brought by motion blur. Nonetheless, the results also reveal limitations: in extreme cases of blur or strong lighting, distal joints such as the ankles or wrists occasionally deviate from their true positions, reflecting the difficulty of capturing fine-grained motion details under severe visual degradation. These findings confirm the effectiveness of MCTN in handling common real-world disturbances, while suggesting potential improvements through integration with temporal modeling or adaptive illumination normalization.

**Table 4:** Comparison with other methods on the MSCOCO validation set.

Methods	Backbone	Frame size	Parameters (M)	AP	AP^50^	AP^75^
TransPose	TransPose-H-A4	256 × 192	17.3	0.753	–	–
SimCC	ResNet-50	256 × 192	25.7	0.708	–	–
HRNet	HRNet-W32	256 × 192	28.5	0.734	0.895	0.807
PRTR	ResNet-50	384 × 288	41.5	0.682	0.882	0.752
EBA	ResNet-18	256 × 256	17.0	0.713	0.915	0.781
RIFormer	HRFormer-B	256 × 192	43.2	0.756	0.908	0.828
BCIR	ResNet-50	256 × 192	34.0	0.675	0.872	0.740
AECA	ResNet-18	384 × 288	19.0	0.745	0.925	0.814
MCTN	DETR	256 × 192	24.5	0.759	0.926	0.822
MCTN	RT-DETRv3	256 × 192	32.8	0.767	0.938	0.836
MCTN	DETR	384 × 288	34.6	0.761	0.922	0.828
MCTN	RT-DETRv3	384 × 288	40.4	0.766	0.941	0.833

#### Comparisons on the MSCOCO dataset

**Fig. 8:**
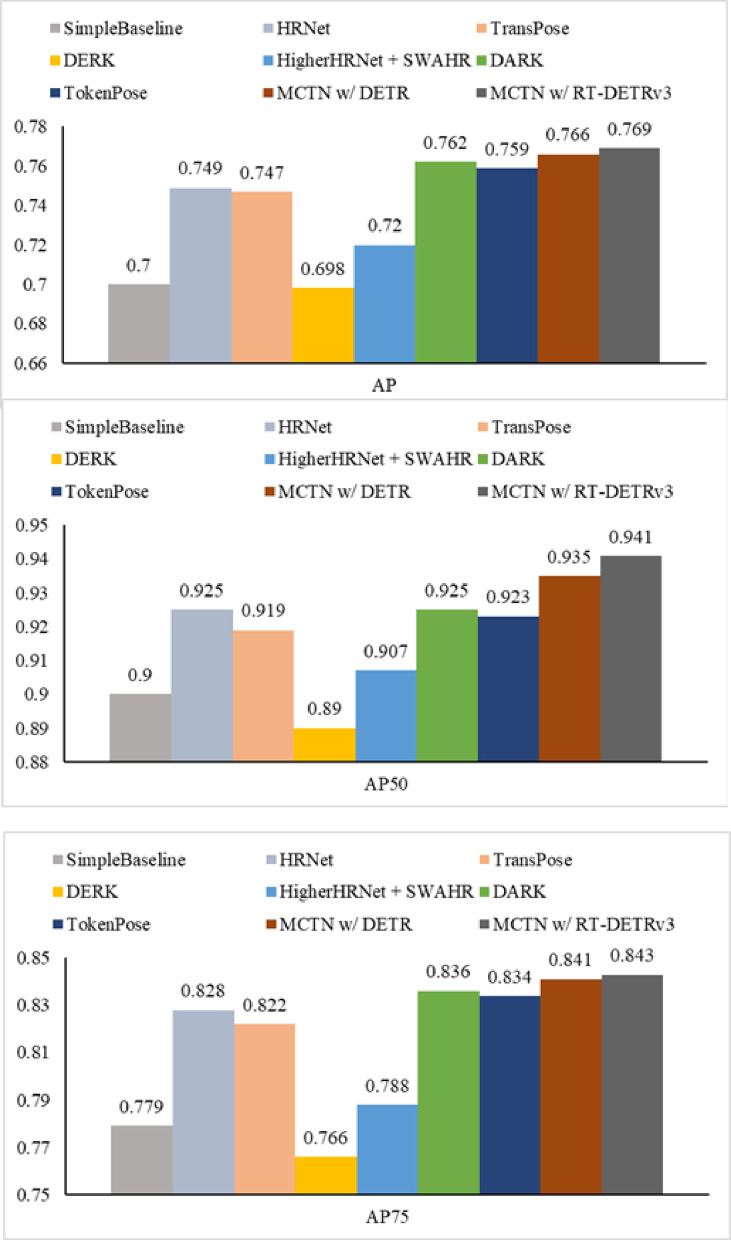
Comparison with other methods on the MSCOCO test set.

To show the stability of MCTN, we conduct extended tests on the MSCOCO Dataset. The experimental results of the Validation set are shown in Table 4. It can be found that MCTN achieves the 0.766 AP score, 0.941 AP^50^ score and 0.833 AP^75^ score on the dataset. Meanwhile, MCTN with different backbones and different input sizes achieves good performance results. The experiments on the test set with the input size of 384 × 288 are shown in Fig. 8. MCTN w/ DETR achieves 0.766 AP score, 0.935 AP^50^ score and 0.841 AP^75^ score. While MCTN w/ RT-DETRv3 achieves 0.769, 0.941, and 0.843 scores on these indicators, respectively.

To more comprehensively reflect the stability and generalization ability of MCTN, we not only conduct extended tests on the MSCOCO Dataset, but also carefully analyze the performance of the model under different configurations. The experimental results on the validation set and the test set clearly show that the MCTN model shows robust performance on this dataset. These results not only verify the advantages of MCTN in dealing with complex scenes and variable target poses, but also highlight its leading position in human keypoint detection tasks. Furthermore, we investigate the different backbone and different input sizes on the performance of MCTN. Experiments present that MCTN can maintain the excellent performance regardless of the backbone or input size, which further proves its strong adaptability and stability.

### Discussion

The empirical evaluation of the MCTN reveals its robust performance in complex human behavior recognition tasks, particularly in scenarios characterized by fast motion, irregular postures, and degraded visual quality. Across both the domain-specific Pickleball dataset and the general-purpose MS COCO dataset, MCTN consistently demonstrates superior accuracy and robustness, validating the effectiveness of its architectural innovations. In high-speed motion contexts, conventional models often fail to capture subtle spatial transformations and lose critical features due to motion blur and occlusion. MCTN overcomes these limitations by introducing a hybrid architecture that integrates three functionally complementary modules—each designed to address a specific visual degradation or recognition bottleneck—thereby improving both the feature discriminability and the model’s generalization ability.

At the module level, the MBR component plays a foundational role by recovering high-frequency texture details that are typically suppressed in blurred sequences. Unlike traditional deblurring approaches, which often rely on handcrafted priors or simplistic convolutional filters, MBR leverages the directional sensitivity and multi-resolution capacity of the curvelet transform to reconstruct motion-degraded inputs with minimal information loss. This facilitates more stable pose estimation and feature encoding in downstream tasks. The MSA module further enhances the model’s ability to adapt to human body variations by allocating attention weights across hierarchical spatial scales. This mechanism not only strengthens the model’s sensitivity to small-scale joint displacements and limb articulations, but also improves its robustness under pose deformation and partial occlusion. Finally, the PCT module introduces frequency-domain semantics into the Transformer architecture, allowing the network to capture structural motion features across orientations and scales. The experimental ablation studies confirm that the inclusion of these three modules leads to measurable performance gains, with each contributing uniquely to the final accuracy, especially in low-quality or cluttered input conditions.

Despite the strong empirical results, several limitations remain that open avenues for future research. First, the incorporation of curvelet-based processing increases the computational overhead of both training and inference stages, which may hinder real-time deployment, especially in resource-constrained environments. Future work could explore model compression techniques or fast approximation algorithms for curvelet transforms to address this challenge. Second, the current model requires high-quality labeled data for optimal performance, yet annotated datasets for fine-grained motion analysis—particularly in niche sports like pickleball—are often scarce and expensive to produce. To alleviate this limitation, future studies may consider semi-supervised or self-supervised learning frameworks that leverage unlabeled data through contrastive or generative mechanisms. Additionally, the static-frame-based architecture of MCTN could be extended to incorporate temporal dynamics through modules such as temporal attention, graph-based spatio-temporal modeling, or recurrent units, thereby enhancing its ability to model continuous motion and behavior evolution in video sequences. These enhancements would further expand MCTN’s applicability in domains such as rehabilitation monitoring, intelligent coaching systems, and real-time interactive environments.

## Conclusion

The MCTN proposed in this study addresses several critical challenges in human behavior recognition from video data, including motion blur, scale variation, and structural complexity of human posture. By incorporating curvelet-based restoration and multi-scale representation into a Transformer framework, the model effectively captures both low-level visual cues and high-level semantic features. The experimental results affirm the advantages of this integrated design, especially in scenarios involving rapid motion and occlusion. Beyond its performance gains, the modular architecture of MCTN offers flexibility for integration with other temporal or multimodal systems. This research not only contributes to the advancement of multi-scale representation learning in vision tasks but also provides a scalable foundation for future development in behavior understanding systems across various domains.

## Supplementary Information

Below is the link to the electronic supplementary material.

Supplementary Material 1

## Data Availability

All data and source codes supporting the findings of this study are available in the supplementary files. (10.3390/sports12090234, 10.1007/978-3-319-10602-1_48).
